# Orbital Mass Detected with POCUS

**DOI:** 10.24908/pocus.v5i1.14222

**Published:** 2020-07-16

**Authors:** Daniel Rusiecki, Andrew Helt, Kathryn McCabe, Colin Bell

**Affiliations:** 1 School of Medicine, Queen's University Kingston, Ontario Canada; 2 Department of Emergency Medicine, Kingston Health Sciences Centre and Queen's University Kingston, Ontario Canada; 3 Department of Emergency Medicine, Denver Health Medical Centre Denver, Colorado United States of America

**Keywords:** POCUS, Ultrasonography, Diagnosis Ophthalmology, Orbital Diseases

## Case File 

**Figure 1:**
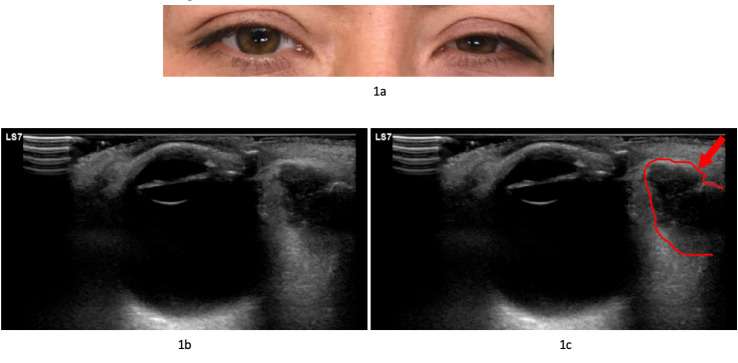
a) Appearance of the patient’s eyes on presentation to the Emergency Department; b) and c) Sonographic appearance of orbit with left lateral rectus muscle. Mass denoted in 1C by red arrow and red outline.

A previously healthy 46-year-old female patient presented to the Emergency Department (ED) with a primary complaint of binocular diplopia worsening over the past 48 hours. Physical exam revealed minor left inferior lid ecchymosis and was significant for proptosis (Figure 1a). There was no pain on extraocular movements, erythema of either lid, induration, chemosis, ophthalmoplegia, relative afferent pupillary defect, or other features of orbital cellulitis. Point of care ultrasound (POCUS) of the globe and orbit was performed and demonstrated a hypoechoic mass within the left lateral rectus muscle (Figure 1b; online Video S1). The patient had orbital CT and MRI imaging revealing, “Two enhancing masses within the left orbit adjacent to or arising from the left lateral rectus muscle causing mild medial displacement of the left optic nerve and mild left proptosis. No evidence of extra orbital extension or perineural spread. Imaging findings nonspecific…”

Orbital masses are rare presentations outside of specialized clinics, and typically involve a comprehensive imaging and laboratory evaluation. Periorbital or orbital cellulitis, thyroid ophthalmopathy, orbital malignancies, systemic autoimmune conditions, and idiopathic orbital inflammation are possible causes of orbital mass 1. Periorbital and orbital cellulitis typically have different physical exam features including discomfort with extraocular motions. On POCUS a periorbital or orbital cellulitis typically has an irregular cobblestone appearance of the edematous subcutaneous tissues with increased echogenicity of the affected tissue 2. Thyroid ophthalmopathy is bilateral in 95% of cases is usually accompanied by laboratory evidence of hyperthyroidism. POCUS findings for thyroid ophthalmopathy include symmetrical thickening of the ocular muscles without discrete mass 3. Finally, orbital malignancy and localized presentations of systemic autoimmune conditions can be expected to have a wide variety of POCUS findings representative of their underlying pathology. 

A diagnosis of idiopathic orbital inflammation (IOI) was suspected based on the clinical history, physical examination and initial imaging findings. IOI, previously known as orbital pseudotumor, is a mass or enlargement in orbital structures as a result of inflammation due to an unknown cause. IOI poses difficulty in diagnosis due to the heterogeneity of clinical presentation, lack of rigid diagnostic criteria, unknown etiology, and similarity to other conditions 4. Thus, it is considered a diagnosis of exclusion once other similarly appearing diseases have been ruled out 5. While there are no sonographic pathognomonic features of IOI, POCUS confirmed the presence of a unilateral asymmetric orbital mass affecting the left lateral rectus muscle, thereby refining the differential diagnosis and streamlining the subsequent advanced imaging studies. 

POCUS is being routinely employed for visualization of intraocular pathology including retinal detachment, vitreous hemorrhage, globe rupture, and intraocular foreign bodies 6. Moreover POCUS is increasingly employed for extraorbital pathology such as optic nerve sheath diameter to assess for intracranial pressure assessment. This case demonstrates that POCUS can be used to not only diagnose intraocular, but also extraocular, intraorbital pathology. The POCUS findings in this case streamlined advanced imaging studies and allowed for rapid oculoplastic surgery referral. 

The patient underwent an orbital mass biopsy performed by the oculoplastic surgery team which may not have obtained a sufficient tissue sample and was ultimately non-diagnostic, being thought not to represent the lesion in question. After observation, serial imaging and repeat ophthalmology follow up for over 1 year, the working diagnosis became orbital venous lymphatic malformation. The sonographic features of orbital venous lymphatic malformations are smooth well circumscribed lesions with high echogenicity 7, 8, and not consistent with the POCUS images obtained. If orbital venous lymphatic malformation is the true diagnosis, the images here may represent a particular subtype or a lesion that matured, subsequently taking on different sonographic findings. The patient’s clinical status has improved; however, a tissue diagnosis has not been obtained.

## Disclosures: 

The authors declare no conflicts of interest. 

## Statement of Consent:

Signed consent from the patient for photographic documentation and publication of the findings was obtained at the time of diagnosis at Denver Health Medical Centre.

## Supplementary Material

Video S1 Dynamic images of left lateral rectus ocular mass on POCUS.
